# *Salmonella* Infections: Global Trends and Emerging Challenges

**DOI:** 10.3390/microorganisms14040816

**Published:** 2026-04-02

**Authors:** Adishi Ranjan, Mahek Chandna, Nicole J. Stevens, Jana Kandil, Brianna Dinh, Macy Kuhn, Noor Mian, Bach Tran, Abdullah Hamid, Peter Kim, Taseen S. Desin

**Affiliations:** 1 College of Medicine, University of Central Florida, Orlando, FL 32827, USAja103381@ucf.edu (J.K.); abdullah.hamid@ucf.edu (A.H.); pe256594@ucf.edu (P.K.); 2 College of Health & Health Performance, University of Florida, Gainesville, FL 32611, USA; 3 Department of Medical Education, College of Medicine, University of Central Florida, 6850 Lake Nona Blvd, Orlando, FL 32827, USA

**Keywords:** nontyphoidal *Salmonella*, type III secretion system, *Salmonella* pathogenicity islands, host-pathogen interactions, antimicrobial resistance, foodborne disease, One Health

## Abstract

*Salmonella* remains a leading cause of foodborne illness worldwide, with non-typhoidal *Salmonella* (NTS) responsible for an estimated 93.8 million infections and substantial global morbidity and mortality. This review synthesizes current evidence on the epidemiology, molecular pathogenesis, and prevention of both typhoidal and nontyphoidal *Salmonella*, with emphasis on emerging challenges in disease control. We highlight key virulence mechanisms, including *Salmonella* pathogenicity islands and Type III secretion systems, that mediate host cell invasion, intracellular survival, and immune modulation, alongside differences in host adaptation, reservoirs, and clinical outcomes between major serotypes. Epidemiological synthesis demonstrates marked geographic variability in disease burden, driven by underreporting, limited diagnostic capacity, and social determinants of health, with particularly high mortality from invasive NTS (iNTS) disease in sub-Saharan Africa. This review further identifies major challenges, including the global rise of multidrug-resistant *Salmonella* lineages, the emergence of high-risk serotypes such as monophasic *S*. Typhimurium and *S*. Infantis, and the increasing complexity of transmission across the farm-to-fork continuum. While advances in whole genome sequencing and integrated surveillance platforms (e.g., PulseNet and GenomeTrakr) have improved outbreak detection and source attribution, gaps in cross-sector coordination persist. Collectively, the evidence underscores the need for integrated One Health approaches that link human, animal, and environmental systems, alongside strengthened surveillance, targeted prevention strategies, and antimicrobial stewardship. Advances in vaccination, including licensed typhoidal vaccines such as Ty21a and Vi polysaccharide, and conjugate vaccines, as well as emerging live attenuated and glycoconjugate candidates targeting NTS, represent promising strategies for reducing the global burden of *Salmonella* infections. Future efforts should focus on improving global surveillance harmonization, addressing environmental and climate-related drivers of transmission, and advancing vaccine development and implementation.

## 1. Introduction

*Salmonella* is a Gram-negative, facultative intracellular, rod-shaped and non-spore-forming member of the Enterobacteriaceae family [1]. The bacterial genus *Salmonella* is divided into two species, *Salmonella bongori* (*S. bongori*) and *Salmonella enterica* (*S. enterica*). *S. enterica* consists of six subspecies: *enterica*, *salamae*, *arizona*, *diarizonae*, *indica* and *houtenae* of which *S. enterica* subspecies *enterica* consists of more than 2400 serotypes [2,3]. Typically, *Salmonella* serotypes are classified into typhoidal (primarily *S. enterica* subspecies *enterica* serovar Typhi) and non-typhoidal serotypes (NTS) (like *S.* Enteritidis, *S.* Typhimurium and *S.* Newport) [4,5,6]. Typhoidal *Salmonella* infections are characterized by typhoid fever and invasive disease, while NTS infections mostly present with self-limiting gastrointestinal disease, which may lead to hospitalization or death. In sub-Saharan Africa, NTS species are known to cause invasive disease (iNTS) that can present as bacteremia with mortality rates approaching 20% [7,8]. Traditionally, NTS infections have been attributed to the consumption of poultry meat and eggs. However, in recent years, fresh produce and other associated food products have been the cause of numerous food-borne outbreaks [9,10]. The global burden for NTS is in the millions, leading to a significant number of deaths, hospitalizations, and massive costs to the health care system [11,12]. Despite increased global surveillance, enhanced detection, and consumer education, NTS remains a public health concern [13].

Given the complexity and continued global burden of *Salmonella* infections, this review aims to synthesize current knowledge on key aspects of *Salmonella* biology and public health impact. Specifically, we summarize the epidemiology and global burden of typhoidal and NTS infections and highlight major virulence mechanisms contributing to pathogenesis. We further discuss emerging public health challenges, including the global rise in antimicrobial resistance, the emergence of high-risk serotypes, and evolving transmission dynamics across the farm-to-fork continuum.

In addition, this review emphasizes a One Health approach by integrating human, animal, and environmental perspectives to better understand *Salmonella* transmission and persistence across interconnected systems. A focused case study examining *Salmonella* transmission and control in pork production systems is included to illustrate the application of a One Health, farm-to-fork framework in a high-risk food production environment. Surveillance and control strategies are then evaluated in the context of current advances in molecular detection, genomic epidemiology, and integrated monitoring platforms. Prevention strategies, including food safety interventions and vaccination approaches, are also reviewed. Collectively, these findings highlight the need for comprehensive and integrated One Health approaches to curb the continued impact of NTS worldwide.

## 2. Global Epidemiological Trends

### 2.1. Salmonella Incidence

#### 2.1.1. Global Incidence and Burden

NTS cases have continued to steadily increase, with a current global case estimate of 93.8 million. Of those cases, there have been 155 thousand reported deaths [14,15]. In the United States, it is estimated that there have been over 1.35 million NTS cases, 26,500 hospitalizations, and 420 deaths [16]. In 2024, salmonellosis was the second most reported zoonotic disease in Europe, with 18.6 confirmed cases per 100,000 people [17]. These estimates differ depending on the region’s surveillance methodology, with some regions only reporting laboratory-confirmed cases (European Union, EU) and others adjusting based on surveys and statistical methods to account for underdiagnosis (U.S.) [18]. Additionally, disease burden is often underestimated in many developing countries due to limitations in diagnostic capacity, including insufficient microbiology laboratories, technicians, and surveillance frameworks. In many African and South American nations, it is estimated that only 1–10% of cases are reported [19]. With many cases being self-limiting or subclinical, they often go unreported when individuals do not seek medical care [20].

Geographic variability in salmonellosis incidence is influenced by a complex interplay of structural, environmental, and healthcare-related factors [21]. Differences in surveillance systems and reporting capacity may contribute to underestimation of disease burden in certain regions, particularly in medically underserved areas with limited access to healthcare and diagnostic services. Environmental and agricultural characteristics also play a role; for example, counties with greater agricultural activity, increased wetland coverage, and impaired surface waterways have been associated with higher salmonellosis incidence. Evidence from a 22-year analysis of Laboratory Enteric Disease Surveillance (LEDS) data (1997–2019), using a counterfactual random forest analysis, further demonstrates that structural inequities, social determinants of health, and environmental factors, including extreme weather, contribute to disparities in salmonellosis incidence across U.S. counties [21]. Additionally, these factors may influence both true disease burden and reporting patterns. These findings underscore the importance of targeted public health interventions, including improved surveillance, enhanced food safety practices, and community-specific prevention strategies.

#### 2.1.2. High-Risk Regions and Vulnerable Populations

Preventing foodborne salmonellosis relies on avoiding cross-contamination and ensuring proper handling of food. This leads to significant variation across regions depending on agricultural practices and food safety regulations. Given the disease’s fecal–oral route of disease transmission, overcrowding, poor water sanitation, and poor hygiene infrastructure contribute to a higher disease burden, leaving low-resource settings particularly vulnerable. This is reflected in regions such as Sub-Saharan Africa, where the death rate for iNTS is 15–25% [13]. Additionally, host risk factors for contracting NTS include advanced age, young children, severe malnourishment, and immunocompromised individuals. Interestingly, there is a particularly significant association between individuals with HIV and their susceptibility to iNTS due to the vital role of Th17 and CD4 T cells in combating *Salmonella* infection [22]. Of the 77,500 deaths in sub-Saharan Africa, 18,400 of those cases were linked to HIV coinfection [9,23].

### 2.2. Trends in Serotypes

#### 2.2.1. Dominant NTS Serotypes

Within the *S. enterica* species, over 2400 non-typhoidal serotypes have been identified worldwide and are classified based on their surface antigens and reactions with antibodies [3,24]. The distribution and prevalence of these serotypes vary by region (Table 1); however, *S*. Typhimurium and *S*. Enteritidis have remained among the dominant serotypes, accounting for over 40% of the total outbreaks in the U.S. [12]. The European Union (EU) reported that the top NTS serotypes from 2018–2021 included *S*. Typhimurium, *S*. Enteritidis, monophasic variant of *S*. Typhimurium (MVST), *S*. Infantis, and *S*. Derby. Moreover, *S*. Enteritidis has remained the dominant EU NTS serotype for decades and has steadily increased from 61.6% to 64.6% of reported cases [25]. Similarly, the aforementioned serotypes have also been reported as the most common serotypes in Africa [26,27]. However, the strain most strongly associated with the region’s high burden of invasive disease is *S.* Typhimurium sequence type ST313, with a large proportion characterized by multidrug resistance (MDR) [27].

#### 2.2.2. Emerging and Re-Emerging Serotypes Worldwide

Aside from the dominant serotypes, several others have emerged and re-emerged, including *S*. Infantis and the MVST. Among these, MVST has been at the forefront of emerging serotypes and is most commonly associated with pork products [28]. It first emerged in Portugal in the 1980s and has since increased in prevalence across the United States and Europe [28]. In 2021, MVST was the most frequently reported serotype in Italy, with 1115 reported cases [28]. Likewise, in Europe, reported MVST cases increased from 1738 in 2023 to 3860 in 2024, making this serotype the third most commonly reported serotype after *S*. Enteritidis [17]. Another emerging serotype is *S.* Infantis; however, unlike MVST, it is predominantly associated with poultry. It is considered the fourth most common serotype in the EU and is frequently associated with multidrug resistance through its megaplasmid known as plasmid of emerging *S*. Infantis (pESI) [29]. It has also recently emerged in the U.S. and Latin America. In a global systematic review (1979–2021), 42.9% of the reported *S.* Infantis isolates were found in the Americas, while 29.8% were found in the EU [30]. More recently, phylogenetic analyses have demonstrated that *S*. Infantis forms a monophyletic lineage that likely originated in West Asia around 1990, followed by spread to Europe, South America, and North America. It has continuously developed more resistance through the expansion of the American sublineage, which has disseminated across all continents. This sublineage includes the extended-spectrum β-lactamase (ESBL)-encoding *bla*_CTX-M-65_ gene, which has further limited treatment options for invasive infections [31].

### 2.3. Outbreak Patterns

#### 2.3.1. Foodborne Versus Non-Foodborne Outbreaks

Recent analyses of outbreak surveillance data from 1998–2022 released by the Interagency Food Safety Analytics Collaboration (IFSAC) state that approximately 75% of human *Salmonella* infections are attributable to foodborne transmission [32]. Some common infected sources from recent multistate outbreaks from 2024–2025 (Table 2) include backyard poultry (1029 total cases; 470 in 2024 and 559 in 2025, an 18.9% increase), eggs (227 total cases; 93 in 2024 and 134 in 2025, a 44.1% increase), and cucumbers (620 total cases; 551 in 2024 and 69 in 2025), all of which may become contaminated at various stages of farming, processing, or food handling. In addition, a charcuterie meat-associated outbreak reported in 2024 resulted in 104 cases [10,33,34].

Common sources for non-foodborne transmissions that are relatively rare but still important include contact with animal feces and reptiles such as pet bearded dragons (26 cases), geckos (49 cases), and small turtles (63 cases) [33]. Other non-foodborne exposures include contaminated water sources and interpersonal transmission, especially in settings such as petting zoos, farms, fairs, schools, and daycares [35]. According to CDC’s Animal Contact Outbreak Surveillance System (ACOSS) data from 2009–2021 (Table 3), 545 enteric disease outbreaks associated with direct or indirect animal contact in the United States resulted in 14,215 illnesses, with *Salmonella* identified as one of the most commonly reported pathogens. Among these outbreaks, 417 had a single reported location of exposure with 6354 illnesses. Of those single-location outbreaks, the most frequently reported settings were private homes (168 outbreaks), public farms or dairies (89 outbreaks), festivals or fairs (36 outbreaks), petting zoos (28 outbreaks), and institutional settings such as schools and daycares (28 outbreaks) [36]. As foodborne and non-foodborne outbreaks intersect with distribution systems that span multiple jurisdictions, the critical role of multistate and multi-country outbreak investigation processes are highlighted.

#### 2.3.2. Multistate/Multi-Country Outbreak Investigations

**Figure 1:**
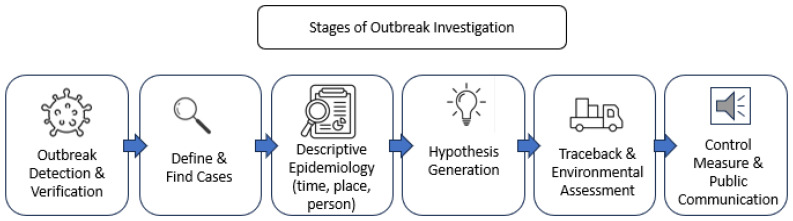
Stages of Outbreak Investigation.

Stages of multistate outbreak investigation are closely parallel to multi-country outbreak investigation frameworks with both grounded in a shared, systematic epidemiologic methodology (Figure 1). The process begins with confirmation that an outbreak is occurring, characterized by an unusual increase in linked cases, followed by verification of the causative agent [37,38]. Standardized case definitions, also known as case count, are developed and refined to clearly specify which individuals meet the criteria for case inclusion [39]. Using this case definition, investigators conduct active case and contact finding through medical records, patient interviews, and fieldwork, and analyze the resulting data by time, place, and person using epidemic curves, geographic mapping, and demographic characteristics to identify patterns of illness [40]. These descriptive findings inform hypothesis generation regarding possible sources of exposure and modes of transmission, which are subsequently evaluated using methods such as case–control or cohort studies [41]. Once a likely source is identified, both frameworks incorporate traceback investigations, laboratory, and environmental testing to determine where contamination may have occurred [40]. Finally, control measures are implemented to limit further transmission, and findings are communicated to public health authorities, regulatory food and veterinary authorities, and the public [42].

#### 2.3.3. Role of Globalization, Travel, and Trade

Globalization has changed food systems through centralized production, international ingredient sourcing, and integrated supply chains, creating the structural framework that enables *Salmonella* to spread beyond local boundaries [43]. Within this structural framework, trade acts as the primary mechanism for dissemination, with evidence from multiple regions, including the United States, China, and Mexico, demonstrating a correlation between increased import–export movement and rising salmonellosis incidence [44,45,46]. International travel further contributes to *Salmonella* outbreaks, with 19% of cases being identified as a major risk factor for high antimicrobial resistance infections, complicating clinical treatment and management [47,48]. A 2019–2023 systematic review that documented multiple disease outbreaks on travel cruise ships, where shared food sources, close living arrangements, and frequent port contact occurred, reported *Salmonella* to be at least one causative agent, as well as Norovirus, *Shigella*, and other pathogens [49]. As the globalization of food systems, trade, and travel continues to contribute to the global *Salmonella* burden, it stresses the importance of continual technological advancements in surveillance systems and collaboration among national and international organizations to link cases across borders.

### 2.4. Surveillance Advances

#### 2.4.1. Whole Genome Sequencing (WGS) and Genomic Epidemiology

A critical step in routine outbreak investigation and food quality control includes the rapid yet sensitive detection of different *Salmonella* serotypes. Whole genome sequencing (WGS) has substantially reshaped surveillance, surpassing other traditional, time-consuming phenotyping methods that can take up to days and weeks [50]. WGS allows for rapid high-resolution tracking and source attribution between closely related isolates in a few hours, with reported overall accuracies >90% and especially strong predictive measures for poultry-associated isolates [51]. The identification of new, emerging, or antimicrobial resistance strains has also been enhanced, supporting more targeted clinical interventions [52]. Adoption of WGS by public health agencies has been a gold standard for many years to build databases that allow for real-time tracking of *Salmonella* infections and collaboration between national and international surveillance platforms, reducing the time needed to investigate outbreaks and enabling faster food recalls and public health decision making [53,54].

#### 2.4.2. Integration of National and International Surveillance Platforms (PulseNet, GenomeTrakr, ECDC, WHO)

Although genomic sequencing provides high-resolution differentiation among *Salmonella* serotypes, its public health impact depends on coordinated international surveillance systems that can translate genomic data into outbreak detection, source attribution, and timely intervention. In the United States, PulseNet, coordinated by the CDC, uses whole-genome sequencing for high-resolution comparison of clinical isolates and real-time outbreak detection, while GenomeTrakr, led by the FDA, complements this effort by linking genomic data from food and environmental isolates to support source tracking and regulatory action [53,55,56]. Beyond these U.S.-based systems, the European Centre for Disease Prevention and Control (ECDC) and the World Health Organization (WHO) play critical roles in expanding surveillance capacity through a broader One Health framework. In the European Union, the ECDC works closely with the European Food Safety Authority (EFSA) under Directive 2003/99/EC to support harmonized zoonotic and foodborne disease surveillance across Member States [17]. Human case data are reported through EpiPulse Cases, while Epi-Pulse enables real-time communication on emerging clusters, and joint ECDC-EFSA molecular databases integrate whole-genome sequencing and other typing methods across human, animal, food, and environmental sources to improve early detection of multi-country outbreaks and source attribution [57,58]. When such outbreaks are identified, ECDC and EFSA issue Rapid Outbreak Assessments to guide interventions and inform policymakers. At the global level, the WHO, in partnership with the Food and Agriculture Organization through the International Food Safety Authorities Network (INFOSAN), provides the primary platform for international food safety communication and emergency coordination, linking hundreds of members across 188 Member States and facilitating rapid alerts when contaminated food enters global trade. INFOSAN also supports emergency preparedness under the International Health Regulations and maintains links with regional alert systems and laboratory networks, including PulseNet International, helping translate laboratory findings into recalls, trade actions, and broader public health responses [53,57,58]. Together, these systems strengthen global *Salmonella* surveillance by integrating human, food, animal, and environmental data, improving detection of multi-state and multi-country outbreaks, and ensuring that endemic regional threats are elevated to international public health attention.

## 3. Molecular Biology and Pathogenesis Updates

### 3.1. Virulence Factor Insights

#### 3.1.1. *Salmonella* Pathogenicity Islands and Type 3 Secretion Systems

With the bacteria’s widespread impact, the *Salmonella* Pathogenicity Islands (SPI) and the Type 3 Secretion System (T3SS) are among *Salmonella*’s major virulence factors, of which SPI-1 and SPI-2 are noteworthy, both of which encode for their respective injectisomes and effector proteins. T3SS-1, encoded by SPI-1, is primarily activated upon contact with the target cell membrane, facilitating the release of effector proteins leading to invasion of the host epithelial cell [59,60,61]. T3SS-2, encoded by SPI-2, plays a major role in intracellular survival via its actions on *Salmonella*-containing vacuoles (SCVs) [59,60,61,62]. Human-derived intestinal epithelial cells cultured with *Salmonella* strains lacking SPI-1 demonstrated a statistically significant decrease in both the mean vacuolar load and percentage of infected cells compared to the wild-type (WT) strain [62]. As for SPI-2, the virulence factor enables the bacteria to survive in the host cells and systematically spread into other organs [59,60,61]. NTS clinical isolates from 200 Saudi Arabian patients in one study detected both SPIs, with 99% being positive for SPI-1 and 84.5% for SPI-2, indicating the epidemiologic importance of these genes [63]. However, SPI-1’s absence in certain human clinical strains (e.g., *S.* Senftenberg) does suggest other mechanisms that still lead to gastroenteritis [64]. 

#### 3.1.2. Adhesion, Invasion, Intracellular Survival Mechanisms

One mechanism for *Salmonella* adhesion is through its fimbriae, as they enable the pathogen to attach onto target cells by interacting with the intestinal epithelial cells’ extracellular matrix (ECM) [65,66]. *Salmonella* also utilizes non-fimbrial adhesins to latch onto enterocytes, with SiiE being notable [67]. Facilitated by a type I secretion system (T1SS) derived from SPI-4, the adhesin binds to cell surface structures consisting of N-acetylglucosamine and α2-3-linked sialic acid [67].

Regarding invasion, *Salmonella* has a plethora of strategies to enter the host cells. The trigger mechanism—mediated by the T3SS-1 effectors—uses multiple proteins to significantly promote and stabilize actin polymerization, resulting in membrane ruffling and bacterial entry [61,66,68,69]. Another method is the zipper mechanism, which utilizes direct contact between *Salmonella*’s Rck and PagN ligands and host cell surface receptors to invoke bacterial internalization [66,68,69]. Other invasion methodology includes the bacteria interacting with microfold (M) cells to undergo transcytosis or hijacking phagocytic cells [60,66,69]. As for *Salmonella*’s intracellular survival, the SCV is a notable component as it shields the bacteria from the host’s defenses by preventing lysosomal fusion [59,60,65].

### 3.2. Host–Pathogen Interactions

#### 3.2.1. Interaction with Intestinal Epithelium, Immune Evasion

As previously stated, the trigger mechanism, mediated by T3SS-1, kickstarts the process with multiple effector proteins, including SipA, SipC, SopB, SopE, and SopE2 [61,66,68]. SipA and SipC are involved in modifying host cells’ cytoskeleton by stabilizing actin via direct binding, with the former further involved by inhibiting actin depolymerization [60,61,66,68,69]. SopB, SopE, and SopE2 promote *Salmonella* invasion by inducing actin remodeling via Rho GTPase family proteins [61,66,68]. The zipper mechanism utilizes the direct binding of *Salmonella*’s Rck and PagN ligands to EGFR and proteoglycan/β-1 integrin, respectively [66,68,69]. Both ligand–receptor interactions ultimately result in actin polymerization, enabling bacterial entry into the intestinal epithelium with minor membrane ruffling [66,68,69]. Another entrance for *Salmonella* is M cells, with *S.* Typhimurium particularly converting follicle-associated epithelial cells into M cells via SopB and exploiting them with ligand–receptor interactions such as transcytosis via FimH/Glycoprotein 2 (GP2) [66,68,69]. Other opportunities involve specific phagocytes such as CXCR1^+^ dendritic cells (DCs) and macrophages, with the bacteria exploiting these cells to cross over the intestinal barrier [61,66,69].

Additionally, *Salmonella* can induce an M2 phenotype in infected macrophages, with the SteE effector driving the process by promoting lower NLRP3 inflammasome activity and allowing for greater survivability [69,70,71]. SpvD can also induce an anti-inflammatory state among the immune cells by blocking the NF-κB mechanism [60,66,70]. SteD is another factor in circumventing host defenses by targeting antigen presentation on major histocompatibility complex (MHC)-II, resulting in less CD4^+^ T cell activation and ultimately immunosuppression [70,72]. Other notable agents involved in immune evasion are SpvC and AvrA, which target MAPK and JNK pathways, respectively, to promote an anti-inflammatory environment [60,66,70,71]. Recent genomic data of 223 clinical *Salmonella* isolates indicates that within-host variation in both virulence and resistance genes (e.g., *emrB*, *sseB*) could ultimately shape the bacteria’s pathogenesis and treatment response [73].

#### 3.2.2. Differences in Virulence Among Major Serotypes

Major *Salmonella* serotypes share a conserved core virulence for epithelial invasion and intracellular survival, but they differ in host adaptation, immune evasion, and accessory genomes, producing substantially different clinical outcomes [74]. According to the U.S. FoodNet data (1996–2006), serotypes differed substantially in invasiveness and clinical severity: *S.* Dublin and *S.* Choleraesuis stood out with very high proportions of invasive infections (blood isolates) and higher hospitalization rates (64% and 57%, respectively) compared with other common gastroenteritis-associated serotypes like *S.* Enteritidis and *S.* Typhimurium [74]. Moreover, *S.* Dublin is disproportionately associated with bloodstream infection, longer hospital stays, and death, characterizing it as a much more invasive serotype [75].

Mechanistically, the major subtypes are divided into typhoidal serotypes (including *S.* Typhi and *S.* Paratyphi) and non-typhoidal serotypes (including *S.* Typhimurium, *S.* Enteritidis, *S.* Dublin, *S.* Choleraesuis, *S*. Heidelberg, *S*. Newport). Typhoidal strains are human-adapted and rely on immune evasion/systemic spread tools such as the Vi capsule and typhoid toxin. The Vi capsule can blunt neutrophil targeting and reshape phagocyte interactions through limiting neutrophil responses while promoting macrophage uptake via DC-SIGN, while the typhoid toxin is an intracellularly produced exotoxin that causes disease [76,77]. Within non-typhoidal salmonellosis, several high-virulence serotypes frequently carry serotype-associated virulence plasmids with the spv operon, which augments intracellular survival and facilitates systemic infection [78]. Interestingly, the survival of *Salmonella* serotypes within the host’s macrophages varies dramatically depending on the host species. For instance, *S.* Typhimurium (a non-typhoidal variant) demonstrated greater survival and replication within mice compared to significant declines in viable counts of *S.* Typhi (typhoidal) strains within mice [79]. Similarly, *S.* Typhimurium induces greater macrophage death in both mouse and human cells compared to each other [79].

### 3.3. Evolution and Adaptation

#### 3.3.1. Genomic Plasticity

The evolutionary success of *Salmonella enterica* is driven by the capacity to gain, lose, and remodel genetic material in response to host and environmental pressures [80]. Comparative genomics of *Salmonella* consistently supports a model in which a relatively conserved core genome is supplemented by a highly variable accessory genome concentrated in regions of genomic plasticity (RGPs), which are regions of a genome that are structurally absent in other similar genomes and are associated with frequent rearrangements facilitated by mobile genetic elements (MGEs) [80]. A large-scale analysis of 12,244 *Salmonella* genomes mapped genome content into regions of genome plasticity, emphasizing how strain-specific acquisition and loss events shape adaptation, pathogenicity, and antimicrobial resistance [80]. Broadly speaking, plasticity is mediated by horizontally acquired genomic islands or pathogenicity islands (PAIs), prophages (which often constitute a major fraction of accessory DNA), conjugative plasmids, and insertion sequences, which drive structural variation [81,82]. Among the best-studied drivers of *Salmonella* plasticity are pathogenicity islands, which are horizontally acquired loci that encode coordinated virulence functions and are frequently associated with tRNA insertion sites, integrases, and atypical GC content [83]. Although many PAIs can be lineage-specific, *S. enterica* broadly shares two cornerstone islands: SPI-1, which encodes effector proteins primarily involved in epithelial invasion, and SPI-2, which encodes effector proteins mainly associated with intracellular survival and replication within host cells [84]. For typhoidal serotypes, larger island-like elements such as SPI-7-related integrative conjugative elements (ICEs) and associated variation have been analyzed as mobile platforms that contribute to typhoid-specific traits and long-term adaptation [85]. Prophages are repeatedly identified as major contributors to *Salmonella* accessory gene content and a major mechanism for rapid diversification. Dedicated reviews emphasize that functional prophages are abundant in *Salmonella* genomes and can mediate lysogenic conversion, adding genes that influence fitness, stress tolerance, immune interaction, and virulence [82]. Conjugative and mobilizable plasmids are another principal engine of genomic plasticity in major *Salmonella* strains, particularly for antimicrobial resistance [86]. Inc-group plasmids (e.g., IncHI2) are frequently implicated in the dissemination of resistance determinants across *Salmonella* species [86]. For example, studies of clinical and food isolates have identified IncHI2 as a prominent plasmid lineage contributing to AMR spread in *Salmonella* species, with multiple MGEs and resistance modules recombining across the plasmid backbone [86].

#### 3.3.2. Environmental Persistence and Stress Resistance

*S. enterica* persists outside the host across a wide range of built and natural environments, including low-moisture foods and processing facilities, farm and animal-production settings, and water/produce-associated niches, because it can rapidly adapt to desiccation, nutrient limitation, temperature shifts, oxidative and acid stress, and sanitizer exposure [87]. In “low-water-activity matrices,” *Salmonella* may not grow, but it can survive for extended periods, creating long-lived contamination reservoirs that are difficult to eradicate once established [87]. *Salmonella* persistence is amplified by surface attachment and biofilm formation on equipment and food-contact materials; biofilms protect cells from dehydration, pH extremes, and antimicrobials and are repeatedly emphasized as a central mechanism for *Salmonella* survival and recurring contamination in food processing environments [88]. Recent experimental data also show *S.* Dublin can survive and even proliferate in sterile bedding sand for days at room temperature, supporting the plausibility of persistence in sterile microenvironments [89].

Mechanistically, stress resistance in major *Salmonella* serotypes reflects both conserved stress-response networks and strain/serotype variation in traits that promote survival on surfaces and in harsh matrices [90]. For long-term environmental survival, extracellular matrix components (ECM components) are strongly implicated; experimental work shows these structures enhance long-term survival and desiccation resistance, making them highly relevant to persistence on dry surfaces and in low-moisture foods [90]. Regarding major *Salmonella* serotypes, it is most accurate to emphasize that many clinically important serotypes (such as *S.* Typhimurium, *S.* Enteritidis, *S.* Newport, *S*. Dublin, etc.) share core stress tools, while differences in persistence often arise from how strongly they form biofilms under relevant temperatures and stressors, how effectively they endure desiccation/low nutrients, and which environments they most frequently cycle through (poultry/eggs, cattle/dairy, produce/water systems), which shapes opportunities for selection and recontamination [90,91].

## 4. Antimicrobial Resistance (AMR) Trends

### 4.1. Global AMR Patterns

#### 4.1.1. Increasing Multiple Drug-Resistant Strains

Although most *Salmonella* infections do not require antibiotic therapy and are managed primarily with rehydration, a significant minority of patients require antibiotics either prophylactically or for the treatment of invasive disease [92]. Prophylactic antibiotic therapy is recommended for patients at increased risk of invasive disease, including immunocompromised individuals and infants younger than three months of age [92]. Additionally, iNTS is characterized by manifestations such as bacteremia, disseminated infection, or enteric fever [92]. In these cases, prompt and effective antibiotic therapy is essential to improving outcomes, particularly among immunocompromised patients, underscoring the ongoing importance of effective antimicrobial treatments for *Salmonella* infections [92]. Over the past several years, there has been a marked global increase in multidrug-resistant (MDR) *Salmonella* strains. Reported MDR prevalence in invasive disease varies substantially across regions, ranging from 45.8% to 75% in invasive disease worldwide [93,94,95]. To improve comparability, these findings were synthesized by geographic region into a structured summary of MDR prevalence estimates, provided in Table 4.

In Europe, a 13-year study in Italy reported MDR in 45.8% of 680 clinical isolates, while data from Romania demonstrated both a high overall prevalence of MDR isolates and a marked temporal increase from 24% to 56% between 2011 and 2021 in poultry-associated strains [96]. In Asia, analysis of 8541 clinical samples in China found that 21.9% of isolates met criteria for MDR, and 69.5% were resistant to at least one antibiotic [97]. In contrast, substantially higher MDR prevalence has been reported in sub-Saharan Africa, where rates have reached approximately 75% among NTS isolates since 2001 [95]. Notably, the high MDR prevalence reported in Romania reflects food-chain isolates from poultry products, which may not be directly comparable to clinical surveillance studies but highlights a significant upstream reservoir contributing to antimicrobial resistance. Although these estimates are derived from heterogeneous study populations and surveillance systems, the consistently high prevalence and overlapping ranges denote a substantial global burden of MDR *Salmonella*, with particularly elevated rates in resource-limited settings.

On the other hand, *S.* Typhi, a serotype primarily associated with typhoid fever in humans and increased mortality rates involving much more severe infection, has also seen large increases in incidence of AMR and MDR serotypes, while an extensively drug-resistant (XDR) strain has become dominant in Pakistan (70% in 2020) and was recently identified in Iraq [98,99]. Although XDR strain dominance has so far been limited to the Middle East, the World Health Organization (WHO), reflecting the growing public health concern, has classified both fluoroquinolone-resistant Typhoidal and NTS as “high-priority” pathogens, the second-highest tier, on its 2024 Bacterial Priority Pathogens List (BPPL), which ranks organisms based on criteria including mortality, disease burden, incidence, and resistance trends over the past decade [99,100]. Although NTS infections are typically self-limiting, this designation underscores the significant and escalating threat posed by increasing MDR *Salmonella* strains [100].

#### 4.1.2. Specific Problematic Lineages

Notably, global surveillance over the past two decades indicates that several of the twenty most prevalent *S. enterica* serotypes have exhibited substantial increases in MDR, including *S.* Typhi, *S.* Infantis, *S.* I 1,4,[5],12:i:-, and *S.* Dublin [93,101]. Among these, the most pronounced increase in MDR prevalence has been observed in *S.* Typhimurium, with MDR rates reaching 73.3% [94,102]. Other serotypes with notable increases in MDR rates include *S.* Enteritidis (42.2%) and *S.* I 1,4,[5],12:i:- (36.0%), as well as *S.* Rissen, which has recently demonstrated resistance to as many as 13 antibiotics [93,102]. In *S.* Typhi, ciprofloxacin resistance has become a global issue, while ceftriaxone and fluoroquinolone resistance continue to increase along with NTS trends [98]. Alongside the rising prevalence of AMR, MDR, and XDR *S.* Typhi serotypes, studies have shown that only 1–4% of patients with suspected typhoid fever in endemic regions such as Asia and Africa have a culture-confirmed *S.* Typhi infection [103]. This low confirmation rate highlights the extensive use of empirical antibiotic therapy and the resulting selective pressure driving antimicrobial resistance in *S.* Typhi [97]. Beyond overall MDR prevalence, AMR trends point to the emergence and persistence of high-risk lineages within several additional serotypes, including *S.* Heidelberg, *S.* Derby, *S.* Muenchen, *S.* Thompson, and *S.* Senftenberg. Of particular concern, fluoroquinolone non-susceptibility has increased markedly in *S.* Enteritidis and *S.* Dublin, further limiting first-line treatment options for invasive infections [93]. Additionally, a particularly concerning MDR lineage of *S.* I 1,4,[5],12:i:- has been identified across Europe, Canada, Australia, and the United States, underscoring its successful international dissemination [104]. This lineage is characterized by resistance to ampicillin, streptomycin, sulfamethoxazole, and tetracycline, a resistance profile that significantly constrains therapeutic options, especially in resource-limited settings [104].

*S.* Infantis represents another high-risk lineage that has undergone a rapid global shift toward multidrug resistance. Once largely antibiotic-susceptible in 2014, the majority of *S.* Infantis isolates displayed MDR phenotypes by 2022 [105]. This transition has been driven in part by a sharp increase in extended-spectrum β-lactamase (ESBL) carriage, with prevalence estimates now approaching 60% [106]. Correspondingly, the incidence of *S.* Infantis infections resistant to five or more antibiotic classes increased from approximately 20% in 2010 to 80% in 2020 [106]. In Peru, iNTS surveillance revealed that 100% of *S.* Infantis isolates were ESBL producers, accompanied by a significant rise in the overall incidence of human disease caused by this serotype [107]. These findings align with trends observed in the United States, where *Salmonella* infections in 2006–2008 were dominated by *S.* Typhimurium and *S.* Heidelberg, followed by declines in these serotypes and a concurrent increase in infections attributable to *S.* Infantis [108].

Another emerging high-risk lineage is *S.* Newport REPJJP01, which was first identified as MDR in 2016 and continued to expand during the COVID-19 pandemic, despite an overall decline in *Salmonella* infections. By 2022, MDR prevalence in this lineage had reached 86% [109]. Importantly, this expansion has been associated with increased clinical severity, as 33% of individuals infected with *S.* Newport REPJJP01 required hospitalization, compared with 27% among patients infected with non-Newport NTS strains during the same period [109].

### 4.2. Mechanisms of Resistance

#### 4.2.1. Plasmid-Mediated Resistance

Plasmids are extrachromosomal DNA molecules within a bacterial cell that are key mediators in horizontal gene transfer. Due to this, plasmid-mediated antimicrobial resistance plays a significant role in the emergence and dissemination of MDR *Salmonella* worldwide [110]. Plasmids are usually classified into incompatibility (Inc) groups, in which all members within the same group cannot be stably maintained within the same bacterial cell line over successive generations [110]. Plasmids of different Inc groups differ in their size, transfer mechanism, and even their antibiotic resistance genes [111]. Multiple plasmid incompatibility groups, including IncA/C, IncF, IncHI, and IncI1, have been strongly associated with antimicrobial resistance in *Salmonella* isolated from humans and animals [111].

Additionally, plasmids in *Salmonella* can have β-lactamase genes, including ESBLs and plasmid-mediated AmpC β-lactamases (PABL), which result in the bacterium having resistance to cephalosporins [112]. Notably, while plasmid-mediated AmpC β-lactamases such as CMY-2 were previously reported predominantly in human *Salmonella* isolates in South Korea, their recent detection in pigs highlights an expansion of resistance plasmids [112].

#### 4.2.2. QRDR Mutations

*Salmonella* infections are often treated with fluoroquinolones or extended-spectrum beta-lactams [113]. As a result, there has been a development of plasmid-mediated quinolone resistance (PMQR), which is encoded by *qnr* genes [114]. These *qnr* genes encode pentapeptide repeat proteins, which work to give the bacterium resistance to quinolones by protecting DNA topoisomerase from the inhibitory effect of quinolones [113]. Moreover, quinolone resistance in *Salmonella* is primarily mediated by chromosomal point mutations within the quinolone resistance-determining regions (QRDRs) of genes encoding DNA gyrase (topoisomerase II) and DNA topoisomerase IV [109]. DNA gyrases and DNA topoisomerase IV are encoded by *gyrA* and *gyrB* genes and *parC* and *parE* genes, respectively [115]. Mutations in these QRDRs result in a lower quinolone-binding affinity of the topoisomerase enzymes, which confers resistance to these quinolone drugs [115]. Most commonly, QRDR mutations that confer resistance to quinolone drugs have been identified in a specific region of the *gyrA* gene, between amino acids 67 and 106 [116]. Studies have shown that *gyrA* QRDR mutations into codons Aspartate-87 (62%) and Serine-83 (38%) were found in 105 *Salmonella* strains resistant to nalidixic acid [116].

#### 4.2.3. Carbapenem Resistance Emergence

Carbapenems are a powerful class of broad-spectrum β-lactam antibiotics that are often considered “last-line agents” or “antibiotics of last resort” due to their unique ability to withstand hydrolysis by most β-lactamases [117]. As such, carbapenems are not commonly used for treating *Salmonella* infections. However, there has been an emergence of carbapenem-resistant Enterobacterales (CRE) strains, including *Salmonella*, around the world, albeit it is still considered a rare occurrence [118]. Regardless, this emergence poses a significant public health concern due to the limited therapeutic options available for multidrug-resistant infections aside from carbapenems [119]. Furthermore, carbapenem resistance has been shown to primarily develop through the acquisition of a carbapenemase gene or the loss of porins, the latter of which results in the antibiotic not being able to be transported into the bacterium, thus rendering it nonfunctional [120]. In addition to these two mechanisms, carbapenem-resistant *Salmonella* strains have also been shown to have an AmpC-type β-lactamase or extended-spectrum β-lactamase [120]. As well, the recent identification of carbapenem resistance in various *Salmonella* strains, such as *S.* Mbandaka, highlights the ongoing expansion of resistance across bacterial strains, which raises concern for the future emergence of extensively drug-resistant *Salmonella* [121]. The major mechanisms of antimicrobial resistance in *Salmonella* that were discussed are summarized in Table 5.

### 4.3. Public Health and Clinical Implications

#### 4.3.1. Treatment Challenges

The rising prevalence of antimicrobial resistance in *Salmonella* has important and interconnected consequences for both clinical care and public health systems. Alongside dramatic increases in MDR, the incidence of severe *Salmonella* infections has also risen, creating substantial challenges for clinical management [122]. MDR trends have disproportionately affected first-line antibiotics traditionally used to treat severe and invasive *Salmonella*, markedly narrowing effective treatment options, particularly in resource-poor settings where access to alternative therapies is limited [122]. At the health system level, escalating resistance contributes to longer hospital stays, increased healthcare expenditures, and higher rates of treatment failure and mortality, particularly in iNTS infections, where mortality is approximately 15%, and patient outcomes are increasingly compromised by escalating resistance and diminishing antimicrobial efficacy [95]. Regional differences in resistance trends further reflect disparities in antimicrobial stewardship infrastructure, antibiotic accessibility, and diagnostic capacity. Higher MDR prevalence in sub-Saharan Africa is often associated with widespread empiric antibiotic use and limited surveillance systems, whereas high-income regions continue to face challenges related to healthcare-associated transmission and antibiotic overuse [95,122]. Collectively, these patterns underscore that antimicrobial resistance in *Salmonella* represents not only a microbiological concern but also a systems-level public health challenge requiring coordinated global responses. Historically, *Salmonella* bacteremia—an infection requiring prompt diagnosis and rapid initiation of therapy to prevent fatal outcomes—was effectively treated with ampicillin or trimethoprim–sulfamethoxazole (TMP-SMX). However, widespread plasmid-mediated resistance has rendered both agents largely ineffective across multiple bacteremia–*Salmonella* strains, leading to their removal from recommended treatment regimens [123,124]. As resistance to these agents became entrenched, fluoroquinolones and third-generation cephalosporins, such as ceftriaxone, emerged as preferred therapies and were initially effective against MDR *Salmonella* strains beginning in the 1980s [124].

More recently, increasing resistance to fluoroquinolones and ceftriaxone has substantially eroded the utility of these critical agents. Fluoroquinolone-resistant *Salmonella* has become sufficiently prevalent that the WHO now classifies fluoroquinolone-resistant NTS and Typhoidal *Salmonella* as “high-priority” pathogens, reflecting the growing urgency of this treatment crisis [95,100,124]. Additionally, the urgent need to swiftly treat typhoidal infections has led to massive empiric antibiotic use, which, combined with low culture-confirmed *S.* Typhi infections, has led to a large increase in treatment failures [103]. Ceftriaxone-resistant *Salmonella* infections have now been documented across a wide geographic range, including the United States, multiple European countries, the Middle East, sub-Saharan Africa, South and Southeast Asia, and China, underscoring the global scale of this challenge [124]. Likewise, fluoroquinolone resistance presents a particular obstacle to effective treatment, as these agents were historically favored for their oral bioavailability, affordability, and broad accessibility, especially in low-resource settings [124]. In response to declining fluoroquinolone efficacy, azithromycin has more recently been adopted as a preferred option for serious infections. Alarmingly, resistance to azithromycin has already been reported in multiple serotypes, with one Italian study identifying near-universal resistance (99.4%), suggesting that its role as a frontline therapy for invasive *Salmonella* infections may be short-lived [94,124].

Surveillance data further indicate a marked rise in resistance to ceftriaxone and cefepime over the past five years, alongside increasing prevalence of strains resistant to fluoroquinolones and other third-generation cephalosporins [95]. Although carbapenems have historically been reserved as last-resort agents for severe *Salmonella* infections, emerging resistance to this class has now been documented in multiple strains, raising concern that even these final therapeutic options may be compromised [95]. The continued emergence and international dissemination of highly resistant *Salmonella* lineages therefore represent a major global public health threat, undermining effective treatment of severe *Salmonella* infection and disproportionately affecting vulnerable and under-resourced populations [124]. Addressing this growing crisis will require coordinated efforts, including the development of novel antimicrobial agents, expanded vaccine strategies, and rigorous enforcement of antimicrobial stewardship programs to slow the further spread of resistance [124]. 

#### 4.3.2. Hospital and Community Settings

Hospital-acquired infections (HAIs) have consistently been associated with higher rates of MDR than community-acquired infections (CAIs), a pattern that also holds true for *Salmonella* infections [123,125]. While the overall MDR rate among CAIs is estimated at 62.5%, MDR prevalence in *Salmonella* infections has been reported to reach as high as 75%, underscoring the disproportionate contribution of *Salmonella* to the burden of antimicrobial resistance [125]. In both hospital and community contexts, antimicrobial-resistant and MDR *Salmonella* infections are associated with prolonged hospitalization—ranging from 0.5 to 2.2 additional days—as well as increased treatment costs when compared with infections caused by drug-susceptible strains [123].

The clinical consequences of MDR *Salmonella* are particularly pronounced in hospital settings. During a six-year outbreak of an MDR *S.* Senftenberg strain associated with HAIs, the case fatality rate reached 4.0%, markedly exceeding the global fatality rate of salmonellosis, which is typically below 1% [124]. Similarly, in Brazil, an outbreak involving ESBL-producing *S.* Infantis infected 140 infants in a neonatal unit, illustrating the dangerous convergence of high-level antimicrobial resistance and vulnerable, immunocompromised patient populations in healthcare-associated settings [124]. On the other hand, in the community setting, fluoroquinolone-resistant *S.* Typhi has emerged as the most common bacterial pathogen responsible for CAIs, reflecting the successful spread of resistant strains beyond healthcare environments [94,100]. The concurrent presence and circulation of MDR and AMR *Salmonella* in both hospital and community settings highlights the permeability of these environments and the potential for bidirectional transmission.

Together, the elevated prevalence and clinical impact of MDR *Salmonella* in both hospital and community settings reflect the global expansion of high-risk lineages, highlighting the urgent need for coordinated surveillance, infection control, and the development of novel therapeutic and preventive strategies.

#### 4.3.3. Importance of Stewardship and Surveillance

The continued global rise in antimicrobial resistance underscores the increasing importance of antimicrobial stewardship, which emphasizes the intentional selection of appropriate, narrow-spectrum antibiotics; reduced reliance on empiric therapy when avoidable; and robust surveillance to limit the spread of resistant organisms such as *S. enterica* [126]. Although carbapenems are typically reserved for severe multidrug-resistant infections, the recent identification of carbapenem-resistant *Salmonella* illustrates that resistance can compromise even last-line treatment options [118]. These findings emphasize the need for effective surveillance systems to identify emerging resistance patterns early and guide appropriate clinical and public health responses [127]. Additionally, healthcare professionals play a critical role in antimicrobial stewardship, as they need to prescribe antimicrobials only when clearly indicated and tailor therapy based on susceptibility data [128]. Stewardship and surveillance efforts are critical to preserving antimicrobial efficacy and slowing the advance of resistant bacterial strains.

## 5. One Health Perspectives

The One Health approach is an interdisciplinary framework that recognizes the fundamental interdependence between human, animal, and environmental health. Many bacterial pathogens, including *Salmonella,* are maintained and transmitted across these interconnected systems, making it insufficient to study and control these infections solely through the lens of human disease [129]. Animal reservoirs, food production and agricultural practices, environmental contamination, and antimicrobial use collectively influence the emergence, transmission, and persistence of bacterial infections in human populations. These infections arise from complex transmission pathways involving animal reservoirs, food production systems, and environmental persistence, underscoring the need for integrated, coordinated control strategies across human, veterinary, and environmental health sectors [129].

### 5.1. Animal Reservoirs and Zoonotic Transmission

#### 5.1.1. Poultry, Cattle, Swine, Reptiles, and Pets

Food-producing animals serve as the principal reservoirs for zoonotic *Salmonella*. Poultry, particularly chickens and turkeys, are the most significant contributors to human infection, with serotypes such as *S.* Enteritidis and *S.* Typhimurium frequently identified from both poultry flocks and human clinical cases [129]. Genetic comparisons have shown significant overlap between strains isolated from retail poultry products and those causing human disease, supporting foodborne transmission from poultry to humans [129,130]. Bacterial colonization of the avian gastrointestinal tract typically presents asymptomatically, allowing poultry birds to act as vectors for *Salmonella* without signs of illness, contaminating eggs, meat, and processing environments [129].

Cattle and swine also represent important reservoirs, harboring a wide diversity of *Salmonella* serotypes that can enter the human food supply through beef and pork products [130]. Studies of cattle herds have shown fecal shedding rates ranging from 20% to 50%, with increased shedding during transport and prior to slaughter, induced by high stress [130]. Swine production systems facilitate amplification through high-density housing and shared feed sources. Antimicrobial use in livestock has further contributed to the emergence of MDR *Salmonella* strains, including those resistant to fluoroquinolones and third-generation cephalosporins, which are commonly used to treat invasive infections in humans [131,132].

Non-food animals also contribute to zoonotic transmission, with an emphasis on young children who have these animals as household pets. Reptiles such as turtles, snakes, and lizards frequently carry *Salmonella* as part of their normal intestinal flora. Reptile-associated *Salmonella* strains account for approximately 6% of sporadic *Salmonella* infections in the United States, disproportionately affecting young children [133]. Common indoor pets, including dogs and cats, may become infected through contaminated pet food or raw meat diets and can transmit *Salmonella* to humans within households [26]. This inter-species transmission highlights the fact that *Salmonella* transmission through animals is not solely limited to food production settings but also occurs in domestic environments.

#### 5.1.2. Food Production Chain Dynamics

Although animals serve as the primary source of *Salmonella*, transmission is amplified throughout the food production chain. Contamination may occur at many points during the food production process, including during animal rearing, slaughter, processing, or distribution. Once introduced into processing facilities, *Salmonella* can persist by forming biofilms on equipment and surfaces, protecting the bacteria from disinfectants, allowing for long-term survival and widespread contamination [134]. Additionally, modern centralized food processing systems have increased the risk of widespread outbreaks. The large quantities of food are produced centrally and distributed over the globalized supply chains, encompassing wide geographic areas. A single contaminated product can therefore lead to infections across multiple states. Fresh produce, once considered a low-risk vehicle, has increasingly been implicated in outbreaks due to contamination from animal manure, irrigation water, or wildlife intrusion [134]. Surveillance data indicate that fruits and vegetables accounted for nearly 46% of foodborne illnesses in the United States between 1998 and 2013 [135]. These dynamics emphasize that *Salmonella* control must address agricultural and environmental sources at every stage from production to fork rather than relying solely on food preparation practices.

### 5.2. Environmental Persistence

#### 5.2.1. Water Systems

Environmental reservoirs play an important role in maintaining *Salmonella* outside animal hosts. Surface waters such as rivers, lakes, and irrigation canals can become contaminated through agricultural runoff, wastewater discharge, and wildlife fecal matter. *Salmonella* is capable of surviving for extended periods in aquatic environments, particularly within sediments and biofilms that protect the organism from environmental stress, facilitating repeated contamination of crops and recreational waters [136]. Moreover, waterborne transmission has been increasingly recognized in produce-associated outbreaks. Investigations have identified contaminated irrigation water as a direct source of *Salmonella* on leafy greens and other vegetables consumed without cooking [137]. Climate-related factors, including flooding and heavy rainfall, further increase this risk by spreading fecal contamination and overwhelming water treatment systems, redistributing pathogens across agricultural landscapes [138].

#### 5.2.2. Agricultural Runoff

Agricultural runoff is a major pathway by which animal-associated *Salmonella* enters soil and water environments. The use of untreated or inadequately treated animal manure as fertilizer introduces viable bacteria into soil, where *Salmonella* may survive for months depending on temperature and moisture [139]. Runoff from concentrated animal feeding operations (CAFOs) can transport the organism into nearby waterways used for irrigation or recreation, concentrating downstream water sources and crops. These pathways are particularly concerning in regions where livestock density overlaps with produce farming, creating opportunities for cross-sectoral pathogen transfer. The persistence of antimicrobial-resistant *Salmonella* in agricultural environments further compounds the challenge, as resistance genes can be maintained and disseminated through environmental microbial communities and transferred to other bacteria, reinforcing connections between antimicrobial use and resistance patterns in human infection [138,139,140].

#### 5.2.3. Wildlife Reservoirs

Wildlife species, including birds, rodents, and wild mammals, contribute to the environmental maintenance of *Salmonella*. These animals can acquire infection from contaminated environments and disseminate the pathogen through fecal shedding across farms and natural habitats. Migratory birds, in particular, have been implicated in the long-distance spread of certain *Salmonella* serotypes, linking geographically distant ecosystems to one another [141]. Wildlife intrusion into agricultural fields has been associated with produce contamination, reinforcing the need for ecological considerations within food safety frameworks.

### 5.3. Integrated Control Efforts

#### 5.3.1. Cross-Sectoral Surveillance and Communication

Effective control of *Salmonella* requires coordinated multiple-sector surveillance across human health, veterinary, and environmental sectors. Integrated systems such as the U.S. National Antimicrobial Resistance Monitoring System (NARMS) monitor *Salmonella* isolates across clinical infections, retail meats, and food animals, providing critical insights into transmission patterns and resistance trends [132]. WGS has further enhanced outbreak detection and source attribution by allowing precise strain comparison and timely public health interventions. These surveillance efforts are most effective when paired with inter-sectional communication. Rapid data sharing between public health agencies, agricultural producers, and environmental regulators facilitates early outbreak identification and coordinated responses that target upstream sources of contamination rather than focusing only on clinical cases [132].

#### 5.3.2. Lessons from Recent Outbreaks Tied to Agriculture and Environment

Recent *Salmonella* outbreaks illustrate the importance of One Health Approaches. Limitations of isolated treatment approaches that focus on only one part of the transmission pathway are not sufficient for effective, widespread pathogen eradication. Supporting this, a One Health European surveillance mapping study of the *Salmonella* pork meat chain in France demonstrated how transmission and surveillance operate across the farm-to-fork continuum, spanning three interconnected sectors: animal health (farm and transport), food safety (slaughterhouse, processing plant, and retail), and public health (general population) [133]. This work highlighted that, despite the presence of surveillance activities within each sector, fragmentation in data flow, limited harmonization of laboratory methods and case definitions, and gaps in inter-sector communication can delay detection and hinder coordinated response efforts [26]. Multistate outbreaks linked to poultry, beef, and fresh produce have repeatedly demonstrated how failures in animal management, water quality, or environmental controls can precipitate widespread human disease [142,143,144]. Furthermore, analyses of outbreak-associated serotypes demonstrate overlap with strains circulating in food animals and agricultural environments. Preventive strategies such as poultry vaccination, improved manure treatment, water irrigation monitoring, and antimicrobial stewardship have been shown to reduce *Salmonella* prevalence at multiple points along the transmission pathway. Interventions targeting a single sector are insufficient; instead, sustained reductions in *Salmonella* burden require simultaneous improvements in animal husbandry, environmental management, food processing hygiene, and consumer education. By addressing shared risk factors across human, animal, and environmental systems, the One Health framework provides a comprehensive approach for reducing the burden of *Salmonella* infections while supporting food safety and public health [144].

## 6. Prevention & Control of *Salmonella*

*Salmonella* causes disease through two closely related but epidemiologically distinct pathways, and effective prevention depends on recognizing these differences. NTS is most often associated with animal reservoirs and the food production chain, with transmission occurring through contaminated foods, cross-contamination during preparation, and direct animal contact in certain settings [145]. In contrast, typhoidal *Salmonella* is primarily maintained through human reservoirs and spreads via the fecal–oral route, particularly in areas where water quality, sanitation, and hygiene practices are inadequate. Prolonged shedding and chronic carriage can further sustain transmission [146]. Table 6 synthesizes these concepts by contrasting non-typhoidal and typhoidal *Salmonella* across key domains, including reservoir, transmission routes, and the highest-yield prevention strategies, providing a practical framework for translating epidemiology into targeted control measures.

Novel approaches to disease prevention for NTS increasingly rely on advanced biotechnology, including the development of various human vaccine candidates and the integration of genomics technology into public health surveillance. Innovative vaccine platforms are currently in the pipeline, such as iNTS-GMMA, which utilizes outer membrane vesicles to elicit a broad bactericidal antibody response, and glycoconjugate subunits like OSP-rT2544 that offer potential cross-protection against multiple *Salmonella* serovars [33]. Furthermore, research into reverse vaccinology and subunit vaccines targeting conserved proteins like InvH is showing promise in animal models. In tandem with these medical advancements, the use of WGS within surveillance networks like PulseNet has revolutionized outbreak management by allowing for the rapid identification of related bacterial clusters and the precise tracing of contamination sources [33]. However, implementing these sophisticated measures in low- and middle-income countries (LMICs), particularly in high-burden regions like sub-Saharan Africa, presents formidable challenges. These hurdles include high production costs, limited coverage across diverse regional strains, and the logistical difficulty of integrating new vaccines into existing national immunization programs [33]. To enhance the value of public health policy, global strategies must address these barriers while tailoring interventions to local agricultural practices and regulatory frameworks, as traditional food safety measures alone are often insufficient to achieve sustained control in these high-risk environments.

In summary, prevention strategies for *Salmonella* should be aligned with the organism’s reservoir and dominant route of spread. Reducing NTS burden relies on integrated food safety measures across production, processing, and consumer handling, supported by hygiene practices and targeted protections for vulnerable populations [145,146]. Limiting typhoidal *Salmonella* transmission requires improvements in water and sanitation systems, consistent hand hygiene, timely diagnosis and appropriate antimicrobial therapy, and vaccination in endemic areas, among travelers, and during outbreaks when indicated [147,149]. Across both entities, robust surveillance and coordinated outbreak response remain essential to identify sources, implement control measures efficiently, and monitor antimicrobial resistance trends.

## 7. Gaps in Knowledge and Future Research Needs

### 7.1. Global WGS Integration Challenges

Since its inception, WGS has become the gold standard for characterizing microbial genomes and linking genetic variation to clinically and epidemiologically relevant phenotypes [169,170,171]. Over the past two decades, declining costs have eliminated major financial barriers, shifting the primary challenges of global WGS integration from technology to international participation and coordination [171,172,173].

Many countries remain hesitant to engage in global surveillance platforms such as Pathogenwatch, NCBI Pathogen Detection, and GenomeTrakr due to data protection laws, regulatory constraints, and concerns over data ownership and privacy [173,174,175]. This fragmentation limits effective global *Salmonella* surveillance. As WGS becomes standard, cross-country comparability is essential; however, harmonization of typing methods remains inadequate, particularly core genome multilocus sequence typing (cgMLST) and whole genome multilocus sequence typing (wgMLST) [169,173,175,176,177,178]. Additionally, an effective data sharing capability may depend on a decentralized database where improved visualization tools are needed to integrate phylogenetic, geographic, and temporal data at both global and local scales [173,174,175].

### 7.2. Understudied Serotypes

Many non-dominant *Salmonella* serotypes remain poorly characterized despite their potential clinical and public health importance. While surveillance focuses on dominant serotypes such as *S.* Typhimurium, *S.* Newport, and *S.* Enteritidis, understudied serotypes, such as *S.* Rubislaw, *S.* Schwarzengrund, *S.* Braenderup, *S.* Saintpaul, *S.* Muenchen, *S.* Montevideo, and *S.* Weltevreden, exhibit distinct epidemiological and genomic features [179,180,181,182,183,184,185,186]. Also, multiple serotypes were observed coexisting within single environmental water sources, highlighting ecological complexity [179,185,187,188]. The BEAM (Bacteria, Enterics, Amoeba, and Mycotics) Dashboard (Table 7) demonstrates the top 10 most common NTS serotypes over the last two years, with *S*. Enteritidis and *S*. Newport being among the most frequent ones, while four of the listed serotypes are understudied [23,189].

Environmental factors, particularly seasonal rainfall and hydrology, were stronger predictors of *Salmonella* presence and diversity than land use or proximity to agriculture, challenging assumptions about farm-dominated sources [185,187]. Genomic analyses revealed notable serotype-specific traits, including the first report of SPI-10 in *S.* Braenderup, SGI-1 in poultry-associated *S.* Schwarzengrund, and CS54 islands limited to *S.* Saintpaul *and S.* Braenderup [190,191], with *S.* Saintpaul demonstrating low plasmid diversity [190,192]. Moreover, the discovery of previously uncharacterized genetic elements, such as aerobactin biosynthesis genes linked to avian adaptation, underscores major gaps in understanding serotype-specific pathogenicity [190,191]. These findings suggest that understudied serotypes may have unrecognized epidemiological and clinical significance.

### 7.3. AMR Surveillance Gaps

Across multiple studies, AMR in *Salmonella* shows strong but imperfect associations with serotype, sequence type (ST), and source [193,194,195,196,197]. Common serotypes such as *S*. Typhimurium, *S*. Enteritidis, *S*. Weltevreden, *S*. Typhi, and *S*. Rissen frequently exhibit ST-specific lineages, with ST34 emerging as an evolutionarily successful backbone capable of accommodating diverse serotype determinants [195,196,197,198,199]. However, repeated identification of unexpected serotype–ST combinations suggests frequent horizontal gene transfer, decoupling serotype from resistance and virulence profiles [195]. This is particularly concerning for *S*. Typhimurium, which carries the largest documented AMR burden and may serve as a key reservoir for transmissible resistance genes, the full extent of whose spread remains poorly understood, especially under selective pressures such as serotype-specific vaccination [94,191,195,196,197,198,199].

Resistance patterns vary substantially by antimicrobial class, host source, and time [197,198,200]. Tetracycline resistance was consistently high across studies, peaking at 77.3% in some datasets, with sulfonamide and streptomycin resistance also commonly reported [190,192,195,197,199,201,202]. Ampicillin resistance generally increased over time but showed regional declines in more recent years [94,195,198]. Fluoroquinolone resistance, including ciprofloxacin, doubled by 2020 before declining in 2022; however, multiple animal-associated studies report substantial ciprofloxacin and azithromycin resistance that is not consistently captured in routine surveillance systems [94,195,198]. Resistance to historical first-line drugs such as chloramphenicol and cephalosporins remains rare in some clinical datasets; it is markedly higher in poultry and livestock isolates [195,198].

Source attribution is another major factor associated with AMR surveillance gaps. More than 50% of resistant isolates originated from food and animal feed sources, with AMR prevalence often exceeding that observed in human clinical isolates [38,190,191,197]. Resistance genes such as *sul1*, *tetA*, and *tetR* were frequently detected even in production settings where antimicrobials were reportedly not administered, suggesting environmental persistence and indirect selection pressures. Emerging MDR lineages, including a novel *S*. Montevideo cluster (ST-10844) with approximately 80% MDR prevalence in China, underscore the growing threat posed by understudied serotypes that fall outside dominant surveillance targets [35,94,197].

## 8. Conclusions and Future Directions

*Salmonella* remains a pervasive global public health threat, characterized by a massive burden of illness and significant mortality across both typhoidal and non-typhoidal serotypes. The pathogen’s remarkable ability to adapt through genomic plasticity and persist in diverse environmental reservoirs underscores the necessity of a One Health approach, which integrates human, veterinary, and environmental health sectors to disrupt complex transmission pathways.

While technological advancements such as WGS have revolutionized outbreak detection and source attribution, significant challenges remain regarding international data-sharing integration and the characterization of understudied serotypes. Furthermore, the escalating prevalence of MDR and XDR strains severely limits traditional treatment options, making robust antimicrobial stewardship and the development of novel therapeutic or vaccine strategies urgent priorities. Ultimately, sustained reductions in the global *Salmonella* burden will require continued cross-sectoral collaboration, enhanced surveillance harmonization, and a deeper understanding of the ecological and molecular drivers of this resilient pathogen.

## Figures and Tables

**Table 1:** Summary of dominant and emerging NTS *Salmonella* strains.

Serotype	Geographic Spread	Epidemiological Status
*S.* Typhimurium	U.S., EU, Africa	Dominant
*S.* Enteritidis	U.S., EU, Africa	Dominant
MVST	EU, U.S.	Emerging
*S.* Infantis	EU, U.S., Latin America	Emerging
*S.* Derby	EU	Dominant
*S.* Typhimurium ST313	Africa	Regionally dominant; recently emerged

**Table 2:** Foodborne and Non-Foodborne *Salmonella* Transmission Types: Reported Case Counts & Hospitalizations from Recent CDC Multistate Outbreaks (2024–2025).

Transmission Type	Subcategory	Example Source	Reported cases	Hospitalizations
Foodborne	Poultry	Backyard poultry	1029 cases	167
	Produce	Cucumbers	620 cases	155
	Egg	Shell eggs	227 cases	72
	Processed meats	Charcuterie	104 cases	27
Non-Foodborne	Reptile contact	Bearded dragons	26 cases	10
		Geckos	49 cases	4
		Small turtles	63 cases	28

**Table 3:** Single-Location Animal Contact Outbreaks and Associated Illnesses: ACOSS, US 2009–2021.

Transmission Type	Animal-Contact Example Settings	Outbreaks, No. (%)	Illnesses, No. (%)
Non-Foodborne	Private homes	168 (40)	3869 (61)
	Farms or dairies	89 (21)	580 (9)
	Festivals or fairs	36 (9)	557 (9)
	Petting zoos	28 (7)	340 (5)
	Institutional settings (school, camp, daycare)	28 (7)	280 (4)
	Other settings not listed	68 (16)	728 (11)
	Total	417	6354 (100)

**Table 4:** Prevalence of multidrug-resistant (MDR) NTS *Salmonella* in clinical and food-chain isolates across regions. Confidence intervals are shown where sufficient data were available. NR = not reported; 95% confidence intervals were calculated using the normal approximation to the binomial distribution.

Region/Country	MDR Prevalence (%)	95% CI (%)	Sample Size (N)	Author
Italy (NTS clinical isolates)	45.8%	42.1–49.5%	680	[94]
Romania (overall, poultry isolates)	85.7% (trend: 24% → 56%)	78.5–92.9%	91	[96]
China (NTS clinical isolates)	21.9%	21.0–22.8%	8541	[97]
Sub-Saharan Africa (invasive NTS)	~75%	NR	NR	[95]

**Table 5:** Summary of the major antimicrobial resistance mechanisms in *Salmonella enterica*, including plasmid-mediated resistance, QRDR mutations, and carbapenem resistance.

Mechanism	Key Genes/Target	Mechanism of Resistance	Antibiotic Class Affected
Plasmid-mediated Resistance	β-lactamases (ESBLs), AmpC (CMY-2)	Horizontal gene transfer of resistance genes via plasmids to prevent the enzymatic degradation of β-lactams	β-lactam Antibiotics
Plasmid-mediated Quinolone Resistance (PMQR)	*qnr* genes	Protection of DNA topoisomerase from quinolone inhibition	Fluoroquinolones
QRDR Mutations	*gyrA* (Ser83, Asp87), *gyrB*, *parC*, *parE*	Reduced quinolone binding due to mutations in DNA gyrase and topoisomerase IV	Fluoroquinolones
Carbapenem Resistance (carbapenemase)	Carbapenemase genes	Enzymatic degradation of carbapenems	Carbapenems
Carbapenem Resistance (porin loss)	Outer membrane porins	Reduced drug entry into the bacterial cell	Carbapenems

**Table 6:** Key differences in reservoirs, transmission pathways, and prevention strategies for non-typhoidal versus typhoidal *Salmonella* (NTS vs. *S.* Typhi/*S*. Paratyphi).

Domain	Non-Typhoidal *Salmonella* (NTS)	Typhoidal *Salmonella* (*S.* Typhi/Paratyphi)
Primary reservoir	Zoonotic + food chain: poultry, eggs, livestock, reptiles; contaminated animal-derived foods [145,146]	Human-only reservoir (carriers and acutely infected people) [147]
Main transmission	Foodborne (undercooked poultry/eggs, meat), cross-contamination, animal contact (esp. reptiles/chicks), occasionally contaminated produce [37,148]	Fecal–oral via contaminated water/food; person-to-person transmission can occur where hygiene is poor [147,149]
Core prevention lever	Food safety across farm → fork [145]	Safe water + sanitation + hygiene, and vaccination in at-risk settings [147,150]
Farm/animal control	Biosecurity, flock/herd testing, vaccination programs in poultry, where used, feed/water hygiene, rodent/insect control, slaughterhouse controls [151,152]	Not applicable (no animal reservoir) [147]
Food handling (consumer)	Cook thoroughly; avoid raw/undercooked eggs; prevent cross-contamination; handwashing after raw meat/animal contact; refrigerate promptly [153,154,155]	Avoid high-risk foods/water in endemic areas (untreated water/ice, raw produce unless peeled, street foods with uncertain hygiene) [147,149]
Food industry controls	Hazard Analysis Critical Control Point (HACCP), pasteurization (eggs/dairy), processing hygiene, cold chain, contamination monitoring, recall systems [156]	Safe food preparation in institutions; monitoring food handlers; rapid investigation of common-source outbreaks [147,157]
Water & sanitation infrastructure	Helpful but not usually the main driver in most settings [144]	Clean water supply, sewage treatment, latrine coverage, and reducing open defecation [158]
Hand hygiene	Important (kitchen, childcare, animal exposure) [147]	Critical (household/community), especially after toileting and before food prep [147]
Vaccination	No routine human vaccine for general NTS prevention (vaccine development for high-risk populations currently in progress) [159,160]	Yes: typhoid conjugate vaccines (TCV) and other typhoid vaccines in endemic areas, travelers, and outbreak control (where recommended) [147,150]
Case management impact on spread	Most cases are self-limited; avoid unnecessary antibiotics to reduce resistance; focus on hydration and infection control in high-risk settings [34,145,161,162]	Prompt diagnosis and appropriate antibiotics shorten illness and shedding; manage dehydration; infection control to reduce onward transmission [34,147,161]
Chronic carriage management	Not a classic long-term carriage problem like typhoid; focus on outbreak source control and hygiene [160]	Chronic gallbladder carriage can occur → identify/manage carriers (public health follow-up; food handler restrictions; targeted therapy and sometimes surgical evaluation in select cases) [144,147]
Healthcare/long-term care prevention	Standard + contact precautions for diarrhea; environmental cleaning; careful food service practices; protect immunocompromised [163]	Same plus heightened vigilance during clusters; ensure safe water/food; manage suspected cases quickly to prevent institutional spread [163]
Outbreak response priorities	Traceback of contaminated food/animal source; product recalls; kitchen/environment sanitation; public advisories (cook/avoid/return products) [34,154,163]	Rapid case finding, water/food source investigation, WASH (Water, Sanitation, and Hygiene) interventions (chlorination/boil-water advisories), targeted vaccination campaigns when indicated [34,147,158,159,163]
Surveillance	Foodborne illness reporting, laboratory subtyping/whole-genome sequencing, monitoring antimicrobial resistance in human and animal isolates [163,164,165,166,167,168]	Case reporting, lab confirmation, monitoring resistance (e.g., fluoroquinolone/cephalosporin/azithromycin patterns), carrier investigations in recurrent clusters [147,168]
Special populations	Extra prevention for infants, elderly, pregnant, and immunocompromised (food avoidance: raw eggs, unpasteurized dairy; avoid reptile exposure) [145,155]	Travelers, residents of endemic areas, and outbreak settings: emphasize vaccine + strict water/food precautions [147]

**Table 7:** CDC’s Bacteria, Enterics, Amoeba, and Mycotics Dashboard of the top 10 *Salmonella* serotypes (2024–2025) in descending order by total number of isolates as of 27 February 2026 [189].

BEAM Top 10	
Serotype	Number of Isolates
*S.* Enteritidis	25,034
*S.* Newport	11,824
*S.* Typhimurium	9169
*S.* Javiana	6436
I 4, 5 12:i:-	4215
*S.* Infantis	3627
*S.* Braenderup	3394
*S.* Saintpaul	3141
*S.* Muenchen	2576
*S.* Oranienburg	2540

## Data Availability

The original contributions presented in this study are included in the article. Further inquiries can be directed to the corresponding author.
